# A genome-wide test for paternal indirect genetic effects on lifespan in *Drosophila melanogaster*

**DOI:** 10.1098/rspb.2021.2707

**Published:** 2022-05-11

**Authors:** Vinesh Naresh Shenoi, Martin I. Brengdahl, Jaime L. Grace, Björn Eriksson, Patrik Rydén, Urban Friberg

**Affiliations:** ^1^ IFM Biology, Linköping University, 581 83 Linköping, Sweden; ^2^ Department of Biology, Loyola University Chicago, 1032 W. Sheridan Rd., Chicago, IL 60660, USA; ^3^ Unit of Chemical Ecology, Department of Plant Protection Biology, Swedish University of Agricultural Sciences, Sundsvägen 14, Box 102, 230 53 Alnarp, Sweden; ^4^ Department of Mathematics and Mathematical Statistics, Umeå University, 901 87 Umeå, Sweden; ^5^ Computational Life Science Cluster (CLiC), Umeå University, 901 87 Umeå, Sweden

**Keywords:** *Drosophila*, paternal indirect genetic effects, lifespan

## Abstract

Exposing sires to various environmental manipulations has demonstrated that paternal effects can be non-trivial also in species where male investment in offspring is almost exclusively limited to sperm. Whether paternal effects also have a genetic component (i.e. paternal indirect genetic effects (PIGEs)) in such species is however largely unknown, primarily because of methodological difficulties separating indirect from direct effects of genes. PIGEs may nevertheless be important since they have the capacity to contribute to evolutionary change. Here we use *Drosophila* genetics to construct a breeding design that allows testing nearly complete haploid genomes (more than 99%) for PIGEs. Using this technique, we estimate the variance in male lifespan due to PIGEs among four populations and compare this to the total paternal genetic variance (the sum of paternal indirect and direct genetic effects). Our results indicate that a substantial part of the total paternal genetic variance results from PIGEs. A screen of 38 haploid genomes, randomly sampled from a single population, suggests that PIGEs also influence variation in lifespan within populations. Collectively, our results demonstrate that PIGEs may constitute an underappreciated source of phenotypic variation.

## 1. Introduction

Parents primarily influence the phenotype of their offspring through the genes they transmit, but potentially also by choosing and/or altering the environment in which their offspring develop (i.e. parental effects). Since mothers in many species invest substantially in offspring, maternal effects have received considerable interest in the past [1], and research has shown that they can have important evolutionary implications [2–5]. Paternal effects, on the other hand, have traditionally been assumed negligible, in all species but in those where males care for their offspring. This view is now rapidly changing. Recent studies, across a wide range of species where males contribute little more than sperm toward offspring, have documented that various manipulations of a sire's environment can have a substantial impact on offspring phenotypes (reviewed in [6–9]).

Paternal effects in species where fathers do not care for their offspring can occur through several different mechanisms. Broadly, they can be categorized into those where the male phenotype directly influences offspring phenotypes and those where they are mediated through a maternal effect [10]. The first class includes epigenetic alteration of sperm DNA due to methylation and chromatin remodelling, as well as small RNAs and proteins deposited into the sperm cytoplasm [11–14]. Paternal effects mediated through a maternal effect can result from proteins in the seminal fluid or through courtship behaviours and other male phenotypes that influence female investment into offspring [15–22].

While our knowledge of paternal effects in species without paternal care has expanded considerably with respect to those mediated through the environment, our understanding of the extent to which the male genotype indirectly influences the phenotype of offspring in such species is limited (from here on called paternal indirect genetic effects (PIGEs)). However, since most mechanisms through which environmental manipulations result in paternal effects presumably also are controlled by genes, it is reasonable to predict that PIGEs are common. Examples of PIGEs in systems where males do not provide for their offspring include female reproductive investment [23,24], juvenile survival [25], juvenile size [26] and certain physiological and behavioural traits [27].

PIGEs are of interest since their heritable component constitutes an additional source of variation through which selection can cause evolutionary changes, potentially leading to more complex evolutionary trajectories than when selection acts on variation caused by direct genetic effects [2,3,28]. Progress in understanding the prevalence and magnitude of PIGEs in species with no paternal care has partly been limited by their presumed negligible size, but perhaps primarily because few breeding designs allow for their estimation [29,30]. A powerful approach to directly test for PIGEs is to use a set of fathers that are heterozygous for a genomic region, where they differ with respect to one allele while the other allele is shared among all fathers. Any consistent phenotypic differences between offspring inheriting the same allele from the genetically distinct fathers are then evidence for PIGEs. This technique has successfully demonstrated PIGEs from single loci (e.g. [31,32]) and sex chromosomes [25,27]. Using *Drosophila* genetics, we here build on an established cytogenetic cloning technique and construct a crossing scheme that allows for direct estimation of PIGEs across more than 99% of haploid genomes in *Drosophila melanogaster.* We use this method to test for PIGEs on lifespan. To gain high power in a first explorative assay, we screened for PIGEs across four geographically distinct populations, since putative PIGEs presumably are larger among genotypes from different populations than among genotypes from the same population. This assay provided support for PIGEs. In a second assay, we followed up these results with a screen for PIGEs within a population, which also supported that PIGEs influence lifespan.

## 2. Material and methods

### (a) Study populations

We first estimated and compared variance in male lifespan due to PIGEs and paternal total genetic effects (i.e. the sum of paternal direct and indirect genetic effects – from hereon called PTGEs) among four populations of *D. melanogaster*. Three of these populations were originally collected in Africa (Congo [Congo], Zimbabwe [Z53], Benin [Dahomey]) and one in North America (California [LH_M_]) (see the electronic supplementary material for more information on these populations). To follow up the results from this assay, we next tested for PIGEs on lifespan within a population of *D. melanogaster*, originally collected in North America (North Carolina, [Raleigh]), using 38 lines from the *D. melanogaster* genetic reference panel (DGRP) [33]. Throughout the experiments, flies were maintained under constant laboratory conditions (12 : 12 light : dark cycle, 25°C, 60% humidity) and fed a standard yeast- and sugar-based medium.

### (b) Crossing design used to estimate PIGEs and PTGEs

**Figure 1.**
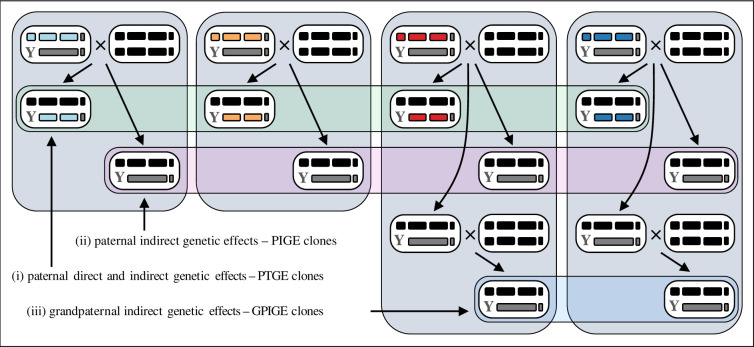
Crossingdesign used to produce males that differ due to (i) the sum of paternal direct and indirect genetic effects (i.e. paternal total genetic effects – PTGEs), (ii) only paternal indirect genetic effects (PIGEs), and (iii) only grandpaternal indirect genetic effects – GPIGEs. Each soft-cornered rectangle filled with white depicts a genotype. Symbols within genotypes show, from left to right: the sex chromosomes (the Y chromosome as the letter Y and the X chromosome as a short bar), the two major autosomes (wild-type copies as long black or coloured bars and a translocation between the major autosomes as a longer gray bar), and the 4th ‘dot’ chromosome (very short bars). To study PIGEs among populations, a single nearly complete haploid genome (coloured) from each of four different populations (LH_M_, Congo, Dahomey, Z53, shown in light blue, orange, red and dark blue colors, respectively) was captured and cloned using the hemiclone technique (see electronic supplementary material, figure S1 for details). These hemiclone males were crossed to virgin females from one population (Dahomey) – crosses in the top of the figure. From each of these crosses two types of sons were produced: those that inherit the two wild-type autosomes from their father (PTGE-clones) and those that inherit the autosomal translocation (PIGE-clones). With no recombination in males, only two other male zygote genotypes are possible (the 4th chromosome was standardized in all hemiclones), both of which are inviable due to aneuploidy. Since the autosomal translocation carries a dominant phenotypic marker (*bw^D^*) PTGE-clone sons and PIGE-clone sons can be separated from each other. Sons inheriting the translocation in all these crosses (PIGE-clones) have the same genotype, despite their fathers having distinct genotypes. Phenotypic differences between PIGE-clones must hence result from the genes not transmitted from father to son. To study PIGEs within a population, 38 hemiclones lines, each carrying an X chromosome and one copy of each major autosome from a DGRP line, were all separately crossed to virgin females from DGRP line 859. To study GPIGEs at the among-population level, PIGE-clone males from two of the populations (Dahomey and Z53), were crossed to Dahomey females (lower crosses in the figure). Sons inheriting the translocation from these crosses (GPIGE-clones) had the same genotype as their fathers, and any phenotypic difference must hence stem from their genetically distinct grandfathers.

Since paternal direct and indirect genetic effects normally co-transmit to offspring, it is difficult to separate out PIGEs using traditional crossing designs. To circumvent this problem, we took advantage of the hemiclone technique developed by William Rice (e.g. [34–39]). This technique allows nearly intact haploid genomes (including the X chromosome and the major autosomes but omits the 4th dot chromosome that comprises less than 1% of the genome) to be captured and cloned, as they are forced to segregate as one unit from father to son. This is made possible through the lack of recombination in males and the use of a translocation between the two major autosomes, in combination with dams carrying a Y chromosome, two attached-X chromosomes and two copies of the autosomal translocation (see electronic supplementary material, figure S1 for details). When crossed to wild-type females, hemiclone males generate two types of gametes that produce viable male zygotes: those that carry the cloned wild-type autosomes and those that carry the autosomal translocation (autosomal aneuploidy makes zygotes formed by the two other possible Y-bearing gametes inviable; figure 1). Since all hemiclone males, irrespective of what wild-type autosomal chromosomes they carry, produce one set of genetically standardized male offspring (with the Y chromosome and the autosomal translocation) when mated to females with a standardized genotype, any consistent phenotypic variation among these male offspring must result from PIGEs (figure 1). From here on, we call these male offspring PIGE-clones. In addition to any PIGEs, male offspring inheriting the distinct wild-type autosomes from their father will vary due to the direct effects of allelic variants residing on these chromosome copies. From here on, we call these male offspring PTGE-clones.

### (c) Experimental procedures

To produce experimental flies for the among-population study, we sampled one haploid genome (X chromosome and the two major autosomes) from each of the four geographically distinct populations and produced one hemiclone with this material per population (electronic supplementary material, figure S1). After amplification, 40 sets of 16 (1–2 days old) males per hemiclone were transferred, without anaesthesia, into vials containing 20 (2–3 days old) virgin wild-type females from the Dahomey population (figure 1). The time allotted for mating was set to 45 min, which should leave enough time for most females to mate once but minimizes the opportunity for extensive courtship and antagonistic interactions between the sexes. Following mating, the flies were lightly anaesthetized and sorted by sex. Males were subsequently discarded while females, in groups of 20, were placed into fresh vials containing standard food medium sprinkled with 8.2 mg live yeast, where they laid eggs for 24 h. We adjusted the number of eggs to approximately 360 in each vial, which corresponds to approximately 180 viable eggs (every second zygote is inviable due to aneuploidy).

Ten days after eggs were laid, we collected two sets of males from each cross: 10 vials with 50 PTGE-clone males and 20 vials with 50 PIGE-clone males. We could separate between the two types of males since the translocation carries *bw^D^*, a dominant phenotypic marker that gives PIGE-clone males brown eyes. Since all focal males had mothers from the same population, no consistent differences among males with different fathers should exist with respect to maternal direct or indirect genetic effects. We chose a higher sample size for PIGE-clone males (1000 per population) than for PTGE-clone males (500 per generation), to increase the power to detect potential PIGEs.

To test if putative PIGEs transmit to grandsons, we created grandpaternal indirect genetic effect clones (GPIGE-clones). These males were produced for two of the populations (Dahomey and Z53), by crossing PIGE-clone sons from the first cross to a new set of virgin Dahomey females (according to the same procedure as above; figure 1). Only sons carrying the shared autosomal translocation originally found in their grandfathers were collected and assayed, since they allowed testing for GPIGEs, including any residing epigenetic marks placed on the Y chromosome and the autosomal translocation by the genetically different haploid genomes residing in grandfathers. For this assay, we collected 20 vials with 50 males from each population.

For the within-population study, we focused exclusively on testing for PIGEs. Focal males for this assay were produced using a procedure similar to the one used in the among-population study (see electronic supplementary material, figure S1). From each of 38 hemiclone lines (each produced from a DGRP line), four sets of 25 males (4 days old) were mated to 25 virgin females from one DGRP line (859; 7 days old), for 60 min. Males were subsequently discarded, and females were transferred to fresh vials. Females were then transferred to fresh vials daily for four consecutive days, and egg numbers were adjusted to approximately 300 per vial (corresponding to approximately 150 viable zygotes). From each of the four sets of four vials per hemiclone, we collected two sets of 50 PIGE-clone males, each separated by one day, summing to eight vials with 50 individuals across four blocks.

To assay lifespan, the flies were transferred into vials with fresh food three times per week (among-population study) or every second day (within-population study). At each transfer, the number of dead flies was recorded. This procedure was continued until all flies were dead. No anaesthesia was used to transfer flies between vials.

### (d) Statistical analyses

All models were fit using Bayesian Hamiltonian Markov chain Monte Carlo via the rstanarm package ([40], v. 2.21.1). For each model, we ran four chains with 4000 iterations each, with the first 1000 iterations discarded as warm-up. Chain convergence was evaluated using the Gelman–Rubin potential scale reduction factor. In order to improve chain convergence when estimating small variance components, the adapt delta was set to 0.99 in all models. For all linear mixed-effect models (LMMs) we used weakly informative, normally distributed priors for both the intercept (mean = 0, s.d. = 10) and the coefficients (mean = 0, s.d. = 2.5), with autoscaling turned on. Variance components were generally calculated from the relevant posterior distributions using the var() function on each iteration of the posterior, but V_R_ was obtained from Sigma posteriors. All statistical analyses were carried out using the R statistical environment (R [41], v. 4.1.1.). All data and code are available on Dryad [42].

To estimate the variance in lifespan due to PTGEs (V_G(D+I)_) among the four separate populations (using PTGE-clone males) we fitted an LMM with the syntax:

Lifespan ∼Population + (1|Vial),

with Population as a fixed effect and Vial as a random effect (accounting for the experimental vial a given fly was housed in). Since variance components are bounded by zero in this model framework we reran the same model 100 times, where in each iteration we randomized the population to which each vial was assigned. We accepted the variance associated with Population as significantly larger than zero, and not an artefact of the sampling algorithm, if the model where vials had their true population identity had a larger estimated variance than at least 95% of the randomized runs [43].

We applied an identical procedure to test for and estimate the variance due to PIGEs among the four populations (V_G(I)_) (using PIGE-clone males), as well as the variance due to GPIGEs using the two populations for which we had data on lifespan for GPIGE-clones. We were not able to detect significant variance in lifespan among GPIGE-clones. To make sure this was not due to a lack of difference in lifespan owing to PIGEs between these particular populations in the parental generation, we reran the analysis for the PIGE-clones only including males from these two populations. To get a more robust comparison we ran the randomized model 1000 times.

In order to compare V_G(I)_ to V_G(D+I)_, we also ran an LMM with the following syntax:

Lifespan ∼Population ∗ Genotype + (1|Vial),

with Population, Genotype (PTGE- or PIGE-clone males) and their interaction as fixed effects and Vial as a random effect.

To test for and estimate the variance in lifespan due to PIGEs within the Raleigh population we fitted an LMM with the syntax:

Lifespan ∼(1|Line) + Block + (1|Vial),

with Line and Vial as random effects and Block as a fixed effect (accounting for the experimental vial and block that a given fly's lifespan was estimated in). To assure that estimated variance components were not an artefact of the sampling algorithm we again reran the model 100 times, where the assignment of each vial was randomized among lines within each block.

## 3. Results

**Figure 2.**
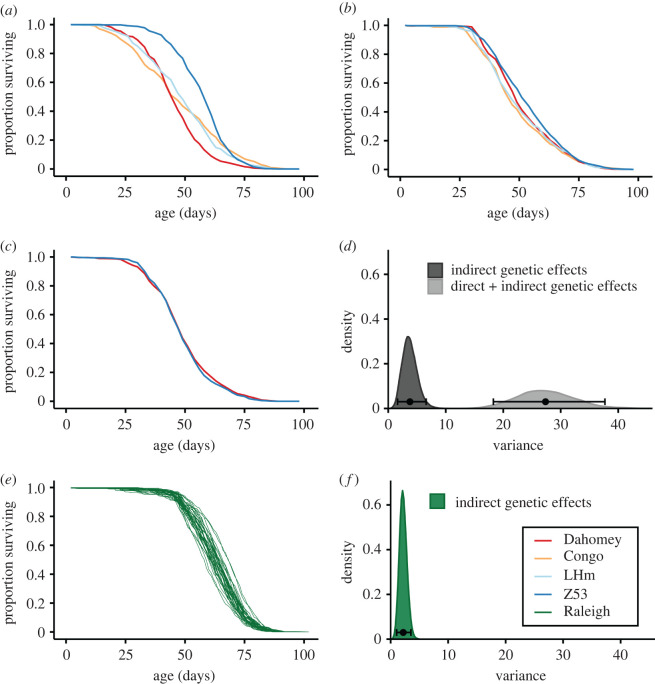
Variation in lifespan due to paternal total genetic effects (PTGEs), paternal indirect genetic effects (PIGEs) and grandpaternal indirect genetic effects (GPIGEs). (*a*) Survival curves for PTGE-clones from the four populations Dahomey, Congo, LHm and Z53. (*b*) Survival curves for PIGE-clones from the four populations Dahomey, Congo, LHm and Z53. (*c*) Survival curves for GPIGE-clones from Dahomey and Z53. (*d*) Posterior distributions, means and 95% CIs for variance in PTGEs and PIGEs among populations. (*e*) Survival curves for PIGE-clones from the 38 DGRP lines (from the Raleigh population). (*f*) Posterior distribution, mean and 95% CI for the variance in PIGEs in the Raleigh population.

At the among-population level, we find evidence for PTGEs as well as PIGEs on lifespan. Analysing PTGE-clones separately we estimate the variance in lifespan (V_G(D+I)_) to 27.10 (Credibility Interval (CI): 19.47–35.83; figure 2*a*; electronic supplementary material, figure S2 and table S1). Using the same approach for PIGE-clones we estimate the variance in lifespan (V_G(I)_) to 3.86 (CI: 1.51–6.97; figure 2*b*; electronic supplementary material, figure S3 and table S1). When analysing PTGE- and PIGE-clone males in the same model, results closely mimic those obtained from the separate models (electronic supplementary material, table S2). With this model we directly compare the sizes of V_G(I)_ and V_G(D+I)_ (figure 2*d*) and find that the former explains 14.2% (CI: 5.63–27.69) of the latter. There was a small difference in average lifespan between PTGE- and PIGE-clone males (mean difference 1.1 days [CI: 0.11–2.10], electronic supplementary material, table S2). When analysing GPIGE-clones we find no evidence for GPIGEs (figure 2*c*; electronic supplementary material, figure S4A and table S1), while their fathers (PIGE-clone males), potentially mediating any such effect, differ significantly in lifespan (figure 2*b*; electronic supplementary material, figure S4B).

We also find evidence for PIGEs at the within-population level, since we detected significant variance in lifespan among the PIGE-clone males generated from the 38 haploid genomes sourced from the Raleigh population (V_G(I)_ = 2.19; CI: 1.05–3.52; figure 2*e*,*f*; electronic supplementary material, figure S5 and table S3). Estimated mean lifespan and CI for all clone lines assayed, at the among- and within-population levels, are reported in electronic supplementary material, table S4.

## 4. Discussion

Using a simple extension of the hemiclone technique, we screened haploid genomes (more than 99%) for paternal indirect genetic effects (PIGEs) in a species where males contribute little more than sperm toward offspring [44]. Our results show that PIGEs contribute significantly to phenotypic variation in lifespan, both among populations and within a population. We find no evidence that PIGEs on lifespan carry over to grand offspring.

To place the estimated variance in lifespan due to PIGEs in context, we compare it to the estimated total amount of genetic variance contributed by fathers to sons at the among-population level. A direct comparison between the estimates we obtained suggests that 14.2% is explained by PIGEs. Although surprisingly high, this estimate is nevertheless biased downward. In nature, fathers influence their offspring's phenotypes via direct genetic effects through the haploid genome they transmit through their sperm (Y chromosome and one copy of each autosome), while their full diploid genomes have the capacity to contribute with PIGEs (Y and X chromosomes and the two copies of each autosome). Since sires in our breeding design only vary with respect to one haploid X and autosomal genome, this underestimates the effect of PIGEs and the proportion of the variance in PTGEs that they explain. Assuming additivity between haploid autosomal genomes and between variance in paternal direct genetic effects (V_G(D)_) and PIGE (V_G(I)_) and noting that the autosomes comprise approximately 80% of the *D. melanogaster* genome, we can correct for this by multiplying V_G(I)_ by 1.8 (1.8 × V_G(I)_ / ([V_G(D+I)_ - V_G(I)_] + 1.8 × V_G(I)_). This provides a new best point estimate of 22.7% (CI: 9.7–40.8). We estimate the variance in lifespan due to PIGEs within the Raleigh population to be slightly more than half of that estimated at the among-population level, but we note that estimates from the two assays are associated with broadly overlapping credibility intervals.

Our separate estimates of variance in PTGEs and PIGEs, as well as the estimated relative size of PIGEs, are associated with a few potential caveats. First, both the Y and the 4th dot chromosomes were standardized in our breeding design, excluding any effect of these chromosomes on either genetic component. The Y chromosome has, however, previously been shown to only have a very small within-population contribution to genetic variance in lifespan in *D. melanogaster* [43], although this effect potentially is larger on the among-population level [45], and the 4th chromosome comprises less than 1% of the genome. There is further no *a priori* reason to believe that any of these chromosomes contribute disproportionally to genetic effects of either kind. Second, our estimates from the among-population assay are based on a limited number of populations, which by chance may have skewed the results in either direction. This includes also potential epistatic interactions between the paternal direct and indirect genetic effects and the maternal genetic background (nuclear and cytoplasmic). Similarly, culturing and assaying flies adapted to partly different environments on the same food source may have caused different degrees of stress and changed the relative magnitude of direct and indirect genetic effects in unpredictable directions. Third, our estimate of the relative contribution of PIGEs assumes that direct and indirect genetic effects act additively. Fourth, a further potential source of bias is that the two types of offspring we produced from each paternal genotype were raised together. We do however note that larval densities were moderate and that both Z53 PTGE-clones and PIGE-clones had the longest lifespans, suggesting no negative interaction between offspring types. Fifth, the fact that we estimate PTGEs and PIGEs in partly different genotypes could have skewed results if there is an interaction between genetic background and PIGEs. And lastly, the variance in PIGEs may be slightly overestimated in relation to the variance in total paternal effects, since the variance tends to scale with the mean and the variance in PIGEs was estimated in a genetic background that on average was slightly more long lived (1.1 days) than the genotypes used to estimate variance due to PTGEs.

The PIGEs we observe were potentially mediated through any or several of a host of different mechanisms. *D. melanogaster* males show genetic variation with respect to components of courtship [46], seminal fluid proteins [47] and the harm they inflict on females [48,49]. This paternal variation may translate into variation in offspring through a maternal effect if it forms the basis for how females adaptively invest [18,50,51] or are coerced to invest [24] in offspring. In line with a maternal effects scenario, it has been found that sire genotype can affect both female gene expression [52] and short-term reproductive investment [24] in *D. melanogaster*. Variation in courtship harm is unlikely to have mediated the effect since we limited dams' exposure to sires to 45 and 60 min in the among- and the within-population assays, respectively (female *D. melanogaster* mate for 15–20 min [53]). An effect of seminal fluids seems more plausible, especially since such effects convincingly have been shown to influence viability in crickets [54,55].

The alternative to a maternally mediated effect is a more direct effect by sires, through small RNAs or proteins deposited in the sperm cytoplasm, or through epigenetic modifications of the DNA transferred to offspring. Since *D. melanogaster* lacks methylation and there is no evidence as yet for paternal effects mediated by sperm loaded with small RNAs or proteins in this species [56], this leaves the possibility that the effect we observe is caused by remodelled chromatin. A previous study has indeed shown that diet-induced changes in chromatin state can affect lifespan in *Drosophila* [57]. While our study did not investigate this option, the lack of variation among grand offspring in the among-population study allows us to conclude that there at least is no evidence for multigenerational effects due to chromatin state [58]. A potential caveat with this conclusion is that the difference in PIGEs between the two populations randomly chosen for this test was small to start with. The lack of variation among grand offspring also suggests that the effect we observe on offspring does not result from differences in germline mutation rates among sires with different genotypes.

We chose lifespan as the readout in our assays, as it is a highly complex trait susceptible to environmental perturbations and influenced by many genes [59]. Lifespan should hence be sensitive to PIGEs if such exist. It is not clear to what extent lifespan is relevant in the laboratory context (especially for populations cultured on short discrete generation cycles), but in the wild it correlates with other life-history traits and shows consistent variation along multiple latitudinal clines (reviewed by [60]). Few studies have tested for PIGEs in general, and we are not aware of any that have addressed their effect on lifespan. Studies investigating the effect of the paternal environment on offspring lifespan are also rare [61], with the one exception of paternal age. In general, these studies show that older fathers have a negative effect on offspring lifespan, but the results span the entire outcome space and are sometimes complex (reviewed in [62]). Studies on *D. melanogaster* [63] and antler flies (*Protopiophila litigata*) [64] found that older fathers in general have longer-lived offspring, while no effect was found in studies of a butterfly [65] and the house sparrow [66]. Negative effects of old fathers have been observed in mice [67,68], the common tern [69] and a neriid fly species [70]. It is possible that the shorter lifespan of offspring to older fathers is explained by the accumulation of mutations in the male germline with age [71], but the age-associated epigenetic changes observed in sperm DNA in mice [68], and the large paternal effect seen across two generations in a fly species [70], suggest paternal effects. Intriguingly, paternal diet and access to food may also influence offspring lifespan, as several studies have found associations between paternal diet and diverse disease phenotypes in offspring [72].

In conclusion, generating genetically identical offspring, using fathers that differ with respect to one allelic copy while they share the other copy, is an efficient way to test for PIGEs. Here, we expanded on this approach and developed a method that allows testing nearly complete haploid genomes for PIGEs and used it to test for PIGEs on lifespan over two generations in *D. melanogaster*. Our findings suggest that PIGEs contribute with a non-trivial component to the total genetic effect that fathers have on their offspring and that they hence could play an important role in the evolution of lifespan. The method opens up the potential for systematic studies of PIGEs in *D. melanogaster*, which should provide us with a better picture of their generality and importance.

## Data Availability

Data and code are available at this link at Dryad Digital Repository: https://doi.org/10.5061/dryad.k6djh9w7q [42]. Extended methods and analyses are provided in electronic supplementary material [73].
